# Crystal structure of (2R*,3aR*)-2-phenyl­sulfonyl-2,3,3a,4,5,6-hexa­hydro­pyrrolo­[1,2-*b*]isoxazole

**DOI:** 10.1107/S2056989016019952

**Published:** 2017-01-01

**Authors:** Yaiza Hernández, Isidro Marcos, Narciso M. Garrido, Francisca Sanz, David Diez

**Affiliations:** a Departamento de Química Orgánica, Universidad de Salamanca, Plaza de los Caidos, 37008 Salamanca, Spain; b Servicio de Difracción de Rayos X, Universidad de Salamanca, Plaza de los Caidos, 37008 Salamanca, Spain

**Keywords:** crystal structure, hydrogen bonds, isoxazolidines, nitro­nes, sulfones

## Abstract

The title compound, C_12_H_15_NO_3_S, was prepared by 1,3-dipolar cyclo­addition of 3,4-di­hydro-2*H*-pyrrole 1-oxide and phenyl vinyl sulfone. In the mol­ecule, both fused five-membered rings display a twisted conformation. In the crystal, C—H⋯O hydrogen bonds link neighbouring mol­ecules, forming chains running parallel to the *b* axis.

## Abstract 2

A new isoxazolidine has been obtained by 1,3-dipolar cyclo­addition of a nitrone and phenyl vinyl sulfone

## Chemical context   

1,3-Dipolar cyclo­addition is one of the most useful reaction in organic synthesis (Pellissier, 2007 ▸). Nitro­nes have been used in the synthesis of many kinds of isoxazolidines (Falkowska *et al.*, 2015 ▸) by 1,3-dipolar cyclo­addition of nitro­nes with sulfones (Flores, García, Garrido, Nieto *et al.*, 2012 ▸) and have demonstrate a range of biological activities including anti­biotic, gene expression regulation and cancer cell cytotoxicity (Karyakarte *et al.*, 2012 ▸). Our research group is inter­ested in the synthesis of isoxazolidines such as the title compound, for application in organic synthesis (Flores *et al.*, 2011*a* ▸,*b* ▸; Flores, García-García *et al.*, 2012 ▸; Flores, García, Garrido, Sanz *et al.*, 2012 ▸; Flores *et al.*, 2013 ▸).
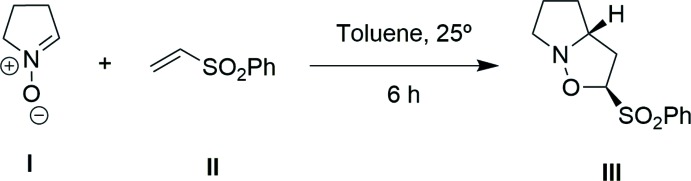


## Structural commentary   

**Figure 1:**
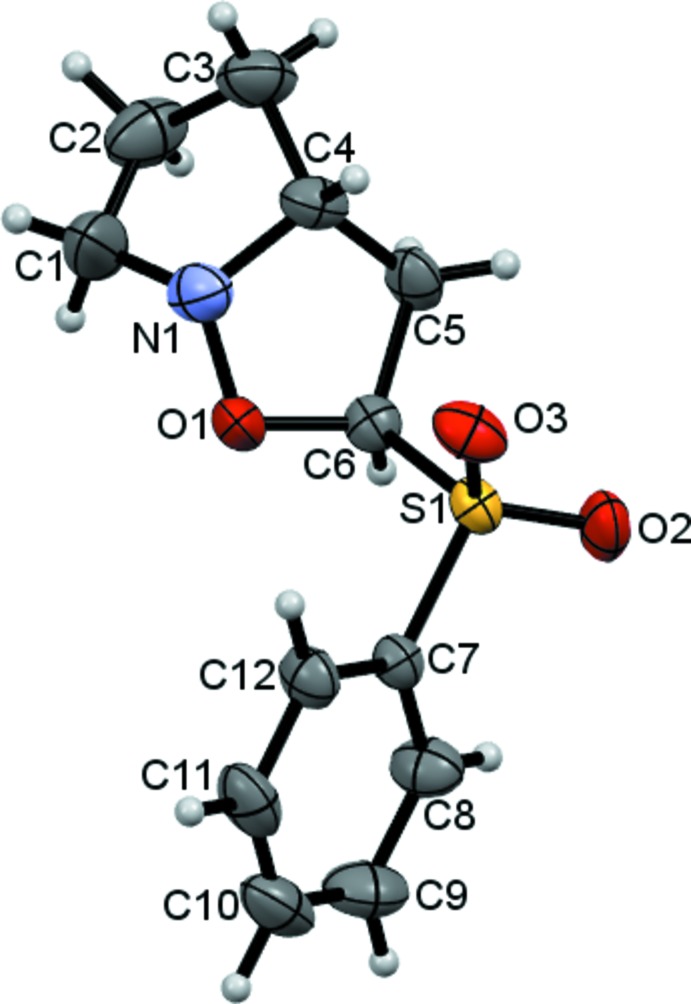
The mol­ecular structure of the title compound, with displacement ellipsoids drawn at the 50% probability level. H atoms are shown as spheres of arbitrary radius.

The mol­ecular structure of the title compound, which consists of an anisoxazol derivative with a phenyl sulfone group as substituent, is shown in Fig. 1 ▸. Both the fused five-membered rings assume a twist conformation, as indicated by puckering parameters *Q* = 0.338 (3) Å, φ = −73.5 (7)° for the pyrrole ring and *Q* = 0.209 (2) Å, φ = −97.5 (6)° for the isoxazole ring. The dihedral angle between the mean planes of the five-membered rings is 64.91 (10)°. All the bond lengths are within normal ranges. The C—S—C and O—S—O angles are 104.34 (9) and 118.54 (11)°, respectively. The large O—S—O angle, and its deviation from the ideal 109.5° angle, can be explained by the repulsion of the lone pairs of the oxygen atoms as far away from each other as possible minimizing the C—S—C angle. The C5—C6—S1—C7 torsion angle is 171.26 (15)°.

## Supra­molecular features   

**Figure 2:**
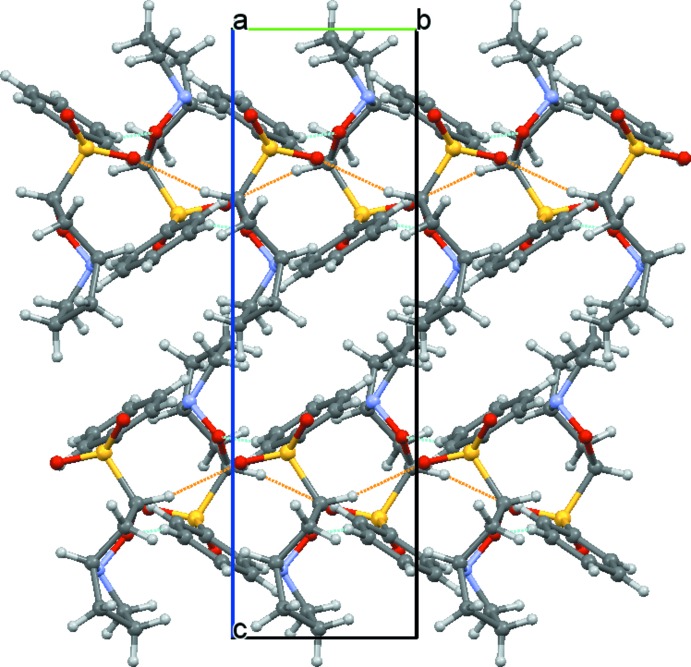
Crystal packing of the title compound viewed along the [100] direction, showing inter­molecular hydrogen bonding (dashed lines).

In the extended structure of the title compound, inter­molecular C—H⋯O hydrogen bonds involving the O1 isoxazole and the O3 phenyl sulfone O atoms as donors (Table 1 ▸) lead to mol­ecular chains running parallel to the *b* axis (Fig. 2 ▸).

## Synthesis and crystallization   

In the synthesis, 5 g of phenyl vinyl sulfone (II) (29.40 mmol) was added to a solution of 2 g of 3,4-di­hydro-2*H*-pyrrole 1-oxide (I) (23.50 mmol) in toluene (75 mL) at room temperature. The resulting mixture was stirred for 6 h, then it was quenched with a saturated aqueous solution of NH_4_Cl and the product was extracted with EtOAc. The combined organic layers were washed with brine, dried over Na_2_SO_4_, filtered, and concentrated, yielding the crude product (III) (8.93 mmol, 38%). The resulting crude residue was purified by flash chromatography (silica gel, hexa­ne/EtOAc 6:4 *v*/*v*) and crystallized from hexa­ne/ethyl acetate solution. IR (film): 3436 (broad), 3068, 2946, 2868, 1442, 1377, 1307, 1148, 1074 cm^−1. 1^H NMR (400 MHz, CDCl_3_, δ p.p.m.): 7.99 (2H, *d*, *J* = 8.0 Hz, H*ortho*), 7.70 (1H, *t*, *J* = 7.9Hz, H*para*), 7.58 (2H, *t*, *J* = 8.0 Hz, H*meta*), 5.04 (1H, *dd*, *J* = 4.0 y 8.4 Hz, H-2), 3.85–3.81(1H, *m*, H-3a), 3.36–3.31 (1H, *m*, H_B_-6), 3.23 (1H, *ddd*, *J* = 4.0, 7.0 y 12.4 Hz, H_B_-3), 3.05 (1H, *dt*, *J* = 8.3 y 13.8 Hz, H_A_-6), 2.50 (1H, *ddd*, *J* = 4.0, 8.4 y 12.4 Hz, H_A_-3), 2.04–1.93 (2H, *m*, H_A_-4 y H_A_-5), 1.76–1.74 (1H, *m*, H_B_-5), 1.60-1.57 (1H, *m*, H_B_-4). ^13^CNMR (100 MHz, CDCl_3_ *δ* p.p.m.): 136.7 (C-*ipso*), 133.9 (CH_*para*_), 129.5 (2CH_*meta*_),128.9 (2CH_*ortho*_), 92.5 (CH-2), 65.5 (CH-3a), 57.3 (CH_2_-6), 36.8 (CH_2_-3), 30.8(CH_2_-4), 23.8 (CH_2_-5). HRMS (EI): C_12_H_15_NO_3_NaS requires (*M*+Na)^+^, 276.0665, found 276.0682.

## Refinement   

Crystal data, data collection and structure refinement details are summarized in Table 2 ▸. The hydrogen atoms were positioned geometrically, with C–H distances constrained to 0.93 Å (aromatic CH), 0.97 Å (methyl­ene CH_2_), 0.98 (methyne CH) and refined using a riding mode with *U*_iso_(H) = 1.2*U*_eq_(C).

## Supplementary Material

Crystal structure: contains datablock(s) global, I. DOI: 10.1107/S2056989016019952/rz5200sup1.cif

Structure factors: contains datablock(s) I. DOI: 10.1107/S2056989016019952/rz5200Isup2.hkl

CCDC reference: 1519195

Additional supporting information: crystallographic information; 3D view; checkCIF report

## Figures and Tables

**Table 1:** Hydrogen-bond geometry (Å, °)

*D*—H⋯*A*	*D*—H	H⋯*A*	*D*⋯*A*	*D*—H⋯*A*
C6—H6⋯O3^i^	0.98	2.35	3.314 (3)	168
C11—H11⋯O1^ii^	0.93	2.49	3.364 (3)	157

Symmetry codes: (i) 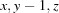; (ii) 
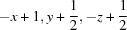
.

**Table 2:** Experimental details

Crystal data	
Chemical formula	C_12_H_15_NO_3_S
*M*_r_	253.31
Crystal system, space group	Monoclinic, *P*2_1_/*c*
Temperature (K)	298
*a*, *b*, *c* (Å)	12.5730 (4), 5.4443 (2), 18.2266 (6)
β (°)	97.754 (2)
*V* (Å^3^)	1236.22 (7)
*Z*	4
Radiation type	Cu *K*α
μ (mm^−1^)	2.31
Crystal size (mm)	0.25 × 0.20 × 0.10

Data collection	
Diffractometer	Bruker APEXII CCD area detector
Absorption correction	Multi-scan (*SADABS*; Bruker, 2006 ▸)
*T*_min_, *T*_max_	0.603, 0.794
No. of measured, independent and observed [*I* > 2σ(*I*)] reflections	9571, 2074, 1949
*R*_int_	0.032
(sin θ/λ)_max_ (Å^−1^)	0.596

Refinement	
*R*[*F* ^2^ > 2σ(*F* ^2^)], *wR*(*F* ^2^), *S*	0.042, 0.109, 1.04
No. of reflections	2074
No. of parameters	154
H-atom treatment	H-atom parameters constrained
Δρ_max_, Δρ_min_ (e Å^−3^)	0.23, −0.25

Computer programs: *APEX2* and *SAINT* (Bruker 2006 ▸), *SHELXS97*, *SHELXL97* and *SHELXTL/PC* (Sheldrick, 2008 ▸) and *Mercury* (Macrae *et al.*, 2006 ▸).

## supplementary crystallographic information

### Crystal data

**Table:**

C_12_H_15_NO_3_S	*F*(000) = 536
*M**_r_* = 253.31	*D*_x_ = 1.361 Mg m^−^^3^
Monoclinic, *P*2_1_/*c*	Cu *K*α radiation, λ = 1.54178 Å
Hall symbol: -P 2ybc	Cell parameters from 1548 reflections
*a* = 12.5730 (4) Å	θ = 8.7–66.1°
*b* = 5.4443 (2) Å	µ = 2.31 mm^−^^1^
*c* = 18.2266 (6) Å	*T* = 298 K
β = 97.754 (2)°	Prismatic, colorless
*V* = 1236.22 (7) Å^3^	0.25 × 0.20 × 0.10 mm
*Z* = 4	

### Data collection

**Table:**

Bruker APEXII CCD area detector diffractometer	2074 independent reflections
Radiation source: fine-focus sealed tube	1949 reflections with *I* > 2σ(*I*)
Graphite monochromator	*R*_int_ = 0.032
phi and ω scans	θ_max_ = 66.8°, θ_min_ = 8.5°
Absorption correction: multi-scan (SADABS; Bruker, 2006)	*h* = −14→13
*T*_min_ = 0.603, *T*_max_ = 0.794	*k* = −6→6
9571 measured reflections	*l* = −17→21

### Refinement

**Table:**

Refinement on *F*^2^	Primary atom site location: structure-invariant direct methods
Least-squares matrix: full	Secondary atom site location: difference Fourier map
*R*[*F*^2^ > 2σ(*F*^2^)] = 0.042	Hydrogen site location: inferred from neighbouring sites
*wR*(*F*^2^) = 0.109	H-atom parameters constrained
*S* = 1.04	*w* = 1/[σ^2^(*F*_o_^2^) + (0.048*P*)^2^ + 0.5869*P*] where *P* = (*F*_o_^2^ + 2*F*_c_^2^)/3
2074 reflections	(Δ/σ)_max_ < 0.001
154 parameters	Δρ_max_ = 0.23 e Å^−^^3^
0 restraints	Δρ_min_ = −0.25 e Å^−^^3^

### Special details

**Table:**

Geometry. All esds (except the esd in the dihedral angle between two l.s. planes) are estimated using the full covariance matrix. The cell esds are taken into account individually in the estimation of esds in distances, angles and torsion angles; correlations between esds in cell parameters are only used when they are defined by crystal symmetry. An approximate (isotropic) treatment of cell esds is used for estimating esds involving l.s. planes.
Refinement. Refinement of F^2^ against ALL reflections. The weighted R-factor wR and goodness of fit S are based on F^2^, conventional R-factors R are based on F, with F set to zero for negative F^2^. The threshold expression of F^2^ > 2sigma(F^2^) is used only for calculating R-factors(gt) etc. and is not relevant to the choice of reflections for refinement. R-factors based on F^2^ are statistically about twice as large as those based on F, and R- factors based on ALL data will be even larger.

### Fractional atomic coordinates and isotropic or equivalent isotropic displacement parameters (Å^2^)

**Table:**

	*x*	*y*	*z*	*U*_iso_*/*U*_eq_	
S1	0.18509 (4)	0.20282 (9)	0.19580 (3)	0.0506 (2)	
O1	0.27595 (12)	0.0779 (4)	0.33002 (8)	0.0780 (5)	
O2	0.10289 (12)	0.0979 (4)	0.14263 (9)	0.0745 (5)	
O3	0.17911 (13)	0.4582 (3)	0.21257 (10)	0.0722 (5)	
N1	0.24857 (16)	0.2358 (4)	0.38806 (12)	0.0767 (6)	
C1	0.2723 (3)	0.0922 (13)	0.45671 (19)	0.173 (3)	
H1A	0.3095	−0.0579	0.4469	0.207*	
H1B	0.3185	0.1863	0.4933	0.207*	
C2	0.1731 (3)	0.0332 (7)	0.48481 (17)	0.1044 (11)	
H2A	0.1821	0.0422	0.5384	0.125*	
H2B	0.1487	−0.1303	0.4696	0.125*	
C3	0.0964 (3)	0.2227 (6)	0.45154 (17)	0.0932 (9)	
H3A	0.1018	0.3717	0.4810	0.112*	
H3B	0.0231	0.1629	0.4466	0.112*	
C4	0.13178 (19)	0.2665 (5)	0.37671 (14)	0.0653 (6)	
H4	0.1134	0.4343	0.3602	0.078*	
C5	0.08854 (17)	0.0856 (5)	0.31715 (13)	0.0669 (6)	
H5A	0.0605	−0.0600	0.3385	0.080*	
H5B	0.0322	0.1592	0.2824	0.080*	
C6	0.18549 (16)	0.0244 (4)	0.27984 (11)	0.0548 (5)	
H6	0.1848	−0.1510	0.2677	0.066*	
C7	0.31162 (15)	0.1448 (4)	0.16793 (11)	0.0489 (5)	
C8	0.3261 (2)	−0.0590 (5)	0.12636 (15)	0.0748 (7)	
H8	0.2699	−0.1680	0.1129	0.090*	
C9	0.4260 (3)	−0.0996 (6)	0.10473 (19)	0.0949 (9)	
H9	0.4371	−0.2375	0.0766	0.114*	
C10	0.5086 (2)	0.0610 (7)	0.12436 (18)	0.0902 (9)	
H10	0.5754	0.0320	0.1095	0.108*	
C11	0.4932 (2)	0.2622 (6)	0.16530 (18)	0.0843 (8)	
H11	0.5495	0.3712	0.1783	0.101*	
C12	0.39431 (18)	0.3066 (4)	0.18785 (14)	0.0650 (6)	
H12	0.3839	0.4444	0.2162	0.078*	

### Atomic displacement parameters (Å^2^)

**Table:**

	*U*^11^	*U*^22^	*U*^33^	*U*^12^	*U*^13^	*U*^23^
S1	0.0409 (3)	0.0501 (3)	0.0601 (3)	−0.00016 (19)	0.0047 (2)	0.0026 (2)
O1	0.0465 (8)	0.1268 (15)	0.0598 (9)	0.0238 (9)	0.0034 (7)	−0.0082 (10)
O2	0.0485 (8)	0.1031 (13)	0.0677 (9)	−0.0149 (8)	−0.0079 (7)	0.0020 (9)
O3	0.0706 (10)	0.0466 (8)	0.1034 (12)	0.0115 (7)	0.0265 (9)	0.0072 (8)
N1	0.0565 (12)	0.0980 (16)	0.0779 (13)	−0.0213 (11)	0.0169 (10)	−0.0249 (12)
C1	0.102 (3)	0.350 (8)	0.0652 (18)	0.079 (4)	0.0086 (18)	0.015 (3)
C2	0.146 (3)	0.098 (2)	0.0722 (17)	0.015 (2)	0.0272 (19)	0.0106 (16)
C3	0.094 (2)	0.111 (2)	0.0831 (18)	0.0098 (18)	0.0415 (17)	0.0043 (17)
C4	0.0646 (14)	0.0611 (13)	0.0743 (14)	0.0105 (11)	0.0250 (11)	0.0033 (11)
C5	0.0437 (11)	0.0849 (16)	0.0728 (14)	−0.0104 (11)	0.0101 (10)	0.0128 (12)
C6	0.0541 (11)	0.0495 (11)	0.0603 (12)	−0.0008 (9)	0.0062 (9)	−0.0008 (9)
C7	0.0446 (10)	0.0478 (11)	0.0539 (10)	−0.0008 (8)	0.0054 (8)	0.0021 (9)
C8	0.0772 (16)	0.0601 (14)	0.0901 (17)	−0.0040 (12)	0.0223 (13)	−0.0145 (13)
C9	0.104 (2)	0.0832 (19)	0.105 (2)	0.0252 (18)	0.0439 (18)	−0.0077 (17)
C10	0.0640 (16)	0.108 (2)	0.105 (2)	0.0248 (16)	0.0352 (15)	0.0315 (19)
C11	0.0447 (13)	0.100 (2)	0.108 (2)	−0.0088 (13)	0.0087 (13)	0.0152 (17)
C12	0.0512 (12)	0.0650 (14)	0.0782 (15)	−0.0091 (10)	0.0064 (11)	−0.0057 (11)

### Geometric parameters (Å, º)

**Table:**

S1—O3	1.4277 (16)	C4—C5	1.511 (4)
S1—O2	1.4364 (16)	C4—H4	0.9800
S1—C7	1.763 (2)	C5—C6	1.511 (3)
S1—C6	1.813 (2)	C5—H5A	0.9700
O1—C6	1.391 (3)	C5—H5B	0.9700
O1—N1	1.440 (3)	C6—H6	0.9800
N1—C4	1.465 (3)	C7—C8	1.370 (3)
N1—C1	1.472 (5)	C7—C12	1.374 (3)
C1—C2	1.446 (5)	C8—C9	1.384 (4)
C1—H1A	0.9700	C8—H8	0.9300
C1—H1B	0.9700	C9—C10	1.367 (5)
C2—C3	1.485 (4)	C9—H9	0.9300
C2—H2A	0.9700	C10—C11	1.354 (5)
C2—H2B	0.9700	C10—H10	0.9300
C3—C4	1.510 (4)	C11—C12	1.382 (4)
C3—H3A	0.9700	C11—H11	0.9300
C3—H3B	0.9700	C12—H12	0.9300
			
O3—S1—O2	118.54 (11)	C3—C4—H4	109.8
O3—S1—C7	108.17 (9)	C5—C4—H4	109.8
O2—S1—C7	109.24 (10)	C6—C5—C4	103.48 (17)
O3—S1—C6	109.58 (10)	C6—C5—H5A	111.1
O2—S1—C6	106.06 (10)	C4—C5—H5A	111.1
C7—S1—C6	104.34 (9)	C6—C5—H5B	111.1
C6—O1—N1	110.65 (15)	C4—C5—H5B	111.1
O1—N1—C4	107.39 (17)	H5A—C5—H5B	109.0
O1—N1—C1	105.4 (3)	O1—C6—C5	107.19 (17)
C4—N1—C1	105.4 (2)	O1—C6—S1	110.63 (15)
C2—C1—N1	109.5 (3)	C5—C6—S1	110.59 (15)
C2—C1—H1A	109.8	O1—C6—H6	109.5
N1—C1—H1A	109.8	C5—C6—H6	109.5
C2—C1—H1B	109.8	S1—C6—H6	109.5
N1—C1—H1B	109.8	C8—C7—C12	120.9 (2)
H1A—C1—H1B	108.2	C8—C7—S1	119.84 (17)
C1—C2—C3	104.2 (3)	C12—C7—S1	119.31 (16)
C1—C2—H2A	110.9	C7—C8—C9	118.8 (3)
C3—C2—H2A	110.9	C7—C8—H8	120.6
C1—C2—H2B	110.9	C9—C8—H8	120.6
C3—C2—H2B	110.9	C10—C9—C8	120.6 (3)
H2A—C2—H2B	108.9	C10—C9—H9	119.7
C2—C3—C4	103.0 (2)	C8—C9—H9	119.7
C2—C3—H3A	111.2	C11—C10—C9	120.1 (2)
C4—C3—H3A	111.2	C11—C10—H10	119.9
C2—C3—H3B	111.2	C9—C10—H10	119.9
C4—C3—H3B	111.2	C10—C11—C12	120.4 (3)
H3A—C3—H3B	109.1	C10—C11—H11	119.8
N1—C4—C3	105.5 (2)	C12—C11—H11	119.8
N1—C4—C5	106.48 (18)	C7—C12—C11	119.2 (2)
C3—C4—C5	115.1 (2)	C7—C12—H12	120.4
N1—C4—H4	109.8	C11—C12—H12	120.4

### Hydrogen-bond geometry (Å, º)

**Table:**

*D*—H···*A*	*D*—H	H···*A*	*D*···*A*	*D*—H···*A*
C6—H6···O3^i^	0.98	2.35	3.314 (3)	168
C11—H11···O1^ii^	0.93	2.49	3.364 (3)	157

Symmetry codes: (i) *x*, *y*−1, *z*; (ii) −*x*+1, *y*+1/2, −*z*+1/2.
